# Patterns of Ultraviolet Radiation Exposure and Skin Cancer Risk: the E3N-SunExp Study

**DOI:** 10.2188/jea.JE20160166

**Published:** 2018-01-05

**Authors:** Isabelle Savoye, Catherine M Olsen, David C Whiteman, Anne Bijon, Lucien Wald, Laureen Dartois, Françoise Clavel-Chapelon, Marie-Christine Boutron-Ruault, Marina Kvaskoff

**Affiliations:** 1 CESP, Fac. de médecine - Univ. Paris-Sud, Fac. de médecine - UVSQ, INSERM, Université Paris-Saclay, Villejuif, France; 2 Gustave Roussy, Villejuif, France; 3 Population Health Department, QIMR Berghofer Medical Research Institute, Herston, Queensland, Australia; 4 School of Public Health, the University of Queensland, Herston, Queensland, Australia; 5 MINES ParisTech, PSL-Research University, O.I.E. – Centre Observation, Impacts, Energy, Sophia Antipolis, France

**Keywords:** melanoma, squamous cell carcinoma, basal cell carcinoma, sun exposure

## Abstract

**Background:**

While ultraviolet (UV) radiation exposure is a recognized risk factor for skin cancer, associations are complex and few studies have allowed a direct comparison of exposure profiles associated with cutaneous melanoma, basal-cell carcinoma (BCC), and squamous-cell carcinoma (SCC) within a single population.

**Methods:**

We examined associations between UV exposures and skin cancer risk in a nested case-control study within E3N, a prospective cohort of 98,995 French women born in 1925–1950. In 2008, a lifetime UV exposure questionnaire was sent to all reported skin cancer cases and three controls per case, which were matched on age, county of birth, and education. Analyses were performed using conditional logistic regression and included 366 melanoma cases, 1,027 BCC cases, 165 SCC cases, and 3,647 controls.

**Results:**

A history of severe sunburns <25 years was associated with increased risks of all skin cancers (melanoma: OR 2.7; BCC: OR 1.7; SCC: OR 2.0 for ≥6 sunburns vs. none), while sunburns ≥25 years were associated with BCC and SCC only. While high-sun protection factor sunscreen use before age 25 was associated with lower BCC risk (*P_trend_* = 0.02), use since age 25 and reapplication of sunscreen were associated with higher risks of all three types of skin cancer. There were positive linear associations between total UV score and risks of BCC (*P_trend_* = 0.01) and SCC (*P_trend_* = 0.09), but not melanoma. While recreational UV score was strongly associated with BCC, total and residential UV scores were more strongly associated with SCC.

**Conclusions:**

Melanoma, BCC, and SCC are associated with different sun exposure profiles in women.

## INTRODUCTION

Ultraviolet radiation (UV) exposure is recognized as a major environmental risk factor for both melanoma and keratinocyte cancers,^1^^–^^6^ two skin cancer types for which the incidence is rising.^7^^,^^8^ However, this factor is difficult to quantify in epidemiological studies: sun exposure is ubiquitous, and individual UV doses vary dramatically depending upon location of residence, time of day and season, pigmentary characteristics, and individual behaviors.^9^ While the influence of UV exposure on skin cancer risk has been well established in ecological studies, epidemiological studies typically fail to show robust associations.^10^ The influence of UV exposures on skin cancer is complex and may differ between skin cancer types.

Nevertheless, the available epidemiological data to date suggest that melanoma and basal-cell carcinoma (BCC) are associated with similar patterns of sun exposure (ie, exposure during childhood and excessive sun exposure in adulthood), particularly in terms of number of sunburns.^3^^,^^11^^–^^17^ In contrast, squamous-cell carcinoma (SCC) risk has been associated with cumulative exposure over the lifetime and occupational sun exposure.^3^^,^^13^^,^^16^^–^^18^ However, while many studies have explored UV exposures in relation to skin cancer risk, most of the previous research compared risk factors across studies conducted separately for melanoma, BCC, and SCC. Very few investigations allowed a direct comparison of skin cancer types within the same study population,^16^^,^^19^ and those studies did not include a comparison according to age at exposure. Moreover, it is currently unclear which spectrum of UV radiation contributes the most to skin cancer development, which is a gap in the literature in this field.^17^

Given the available knowledge, we aimed to quantify the associations between various UV exposures and the risks of melanoma, BCC, and SCC and to examine the patterns that are the most predictive of skin cancer risk in a population of French women. We also aimed to identify the type of UV radiation (UVA, UVB, erythemal UV, total UV) associated with each skin cancer type.

## METHODS

### Study population

E3N (*Etude Epidémiologique auprès de femmes de l’Education Nationale*) is a prospective cohort study involving 98,995 women born in 1925–1950, living in metropolitan France at inclusion, and insured through a national health scheme primarily covering teachers.^20^ Women were enrolled from February 1^st^, 1989, through November 30^th^, 1991 after returning a baseline self-administered questionnaire on their lifestyle and medical history, along with informed consent. Follow-up questionnaires were sent every 2–3 years thereafter. All cohort questionnaires inquired about the occurrence of skin cancers, requesting contact details of the participants’ physicians and permission to contact them to retrieve histological records. The study received ethical approval from the French National Commission for Computerized Data and Individual Freedom.

In 2008, a case-control study nested within E3N, the E3N-SunExp study, was conducted to collect detailed data on UV exposures in a subset of participants. Women eligible for inclusion into the sub-study were those diagnosed with a first primary incident skin cancer up to January 10^th^, 2008 (cases) and women who were free of cancer at that date (controls). For each skin cancer case, three controls were randomly selected using incidence density sampling and were matched according to age, county of birth, and education level. A skin cancer case was defined as the first diagnosis of a primary incident melanoma, BCC, or SCC, whichever occurred first. In the case of concurrent diagnoses of BCC and melanoma or SCC, the case was regarded as melanoma (*n* = 16) or SCC (*n* = 25) over BCC. Histological confirmation was obtained for 99% of melanoma, 95% of BCC, and 96% of SCC cases. A total of 22/460 (4.8%) melanoma cases, 15/1,286 (1.2%) BCC cases, and 4/215 (1.9%) SCC cases died before the study and could not be included as participants.

We collected UV exposure data through a self-completed questionnaire that was modelled on a similar instrument used in previous Australian studies and sent to participants on January 10^th^, 2008.^21^ A reminder was sent to non-respondents on May 25^th^, 2008 (*n* = 2,237). Questionnaires were mailed to 424 melanoma cases, 1,193 BCC cases, 196 SCC cases, and 5,438 matched controls and were successfully returned by 368 melanoma cases, 1,032 BCC cases, 166 SCC cases, and 4,215 matched controls, resulting in response rates of 87% for cases and 79% for controls. Compared with non-respondents, respondents were more likely to be cases than controls (27.1% vs. 16.7%), to be younger, and to have a higher level of education (*p *< 0.001). However, other characteristics (such as pigmentary traits and family history of skin cancer) were similar between the two groups (eTable 1). After excluding cases that had no matched control, our final sample consisted of 366 melanoma cases, 1,027 BCC cases, 165 SCC cases, and 3,647 matched controls.

### Exposure assessment

#### Sunburn, sunscreen use, and indoor tanning

Women were asked to report the number of times they were sunburned so badly that they developed either blistering of the skin, soreness for 2 or more days, or peeling of the skin at different ages: before 15 years old, between 15 and 25 years, and since age 25 years (never, once, 2–3 times, 4–5 times, or ≥6 times). We also collected the sun protection factor (SPF) of the sunscreen that they usually used during each of these periods (no protection, <8, 8–14, 15–30, or >30/total sunblock) and their usual habits of reapplying sunscreen during sun exposure (never, sometimes, or always). Women were additionally asked to report if they had ever used indoor tanning devices, and if so, how frequently they used these devices on average, how many sessions they had in total over their lifetime, and how long they were exposed on average at each session.

#### Lifetime sun exposure

Participants were also asked to complete a lifetime diary of residence locations. For each location, women reported the ages at which they lived in each place, the amount of time they usually spent outdoors in the sun in spring/summer (≤1 hour/day, 2–3 hours/day, or ≥4 hours/day), and their level of sun protection using clothing or sunscreen when in the sun (“never or rarely”, “sometimes”, or “often or always”). We asked these questions separately for work/school days and weekends/days off. Women then completed a similar lifetime diary for holiday locations, where they were additionally asked to report the number of weeks that they spent in each place, along with their time spent in the sun and their level of sun protection. Using these data, we calculated two principal measures of UV exposure: lifetime number of hours of sun exposure, and a UV score (eMethods 1).

In addition to sunburn, which highly reflects intensity of UV exposure, the summary measures that we used allowed exploring both intensity and duration of exposure. While number of hours of sun exposure enabled us to study duration of exposure taking sun protection into account, UV scores included both intensity and duration by combining information on hours of sun exposure and UV dose associated with each place of residence/holiday.

#### Phenotypic factors

Phenotypic information was collected in the baseline questionnaire of the cohort in 1990 and included self-reported information on natural hair color (red, blond, chestnut, brown, or black), skin color (very fair, fair, medium, brown, or black), number of naevi (very many, many, few, or none), number of freckles (very many, many, few, or none), and skin sensitivity to sun exposure (high, moderate, or low). Eye color (blue or grey, green, hazel, brown, or black) was collected through the questionnaire of the E3N-SunExp sub-study.

### Statistical methods

We used conditional logistic regression modelling to calculate odds-ratios (ORs) and 95% confidence intervals (CIs) for the associations between UV exposures and skin cancer risk. ORs were first estimated in crude models (considering matching for age, county of birth, and education), then in models adjusted for phenotypic factors (skin sensitivity to sun exposure, number of naevi, number of freckles, and eye, skin, and hair color). ORs estimating risk associated with the number of sunburns were not adjusted for skin sensitivity to sun exposure. An additional model was further adjusted for family history of skin cancer; however, since the results were identical, we elected to present only those from the first two models. Tests for linear trend were performed using an ordinal score for each factor. Homogeneity tests were performed to test for differences in estimates between the three skin cancer types.^22^ For all variables, multiple imputation of missing values was performed. The data were imputed five times using a fully conditional specification method (SAS procedure PROC MI). The pooled estimate was obtained by averaging the estimates from the five imputed datasets and the confidence intervals took into account within- and between-imputation variances. The results obtained with multiple imputation were compared with those from the complete case analysis (*n* = 2,408); since the results obtained with the two methods were almost identical, we only presented those arising from multiple imputations.

Stratification analyses were conducted for the number of hours of sun exposure and UV scores according to age of exposure (<25 years or ≥25 years) and homogeneity tests were performed to test for differences in estimates across strata.

The main analyses on UV score were conducted using data on total UV, and sensitivity analyses were performed according to the specific type of UV radiation (UVA, UVB, and erythemal UV). All statistical analyses were performed using SAS, version 9.3 (SAS Institute, Cary, NC, USA).

## RESULTS

At the time of the questionnaire, the participants of the E3N-SunExp study were aged 57–85 years old, with a mean age of 68 (standard deviation, 7) years. For all cases, the time-interval between diagnosis and response to the questionnaire ranged from 3 to 18 years. Women with high skin sensitivity to sun exposure were more likely to be diagnosed with skin cancer, especially melanoma and SCC, compared with those with lower sensitivity (eTable 2). Melanoma and BCC cases had higher numbers of naevi and freckles and were more likely than controls to have a fair pigmentary profile: red/blond hair, fair skin, and blue/grey eyes. SCC cases were more likely than controls or other cases to have high numbers of freckles and fair skin.

### Sunburn, sunscreen use, and indoor tanning

We observed positive linear relationships between a history of sunburns before 15 years and at 15–25 years and the risks of melanoma (*P_trend_* = 0.007 and 0.002, respectively) and BCC (*P_trend_* < 0.0001 for both) (Table 1). While strong positive linear associations were also observed for SCC in crude models (*P_trend_* = 0.0003 for both age categories), associations were substantially attenuated after adjustment. Number of sunburns after age 25 was positively associated with BCC and SCC risks, but not with melanoma risk, although there was no heterogeneity across skin cancer types (*P_homogeneity_* = 0.60).

**Table 1.** Crude and adjusted ORs for risks of melanoma, BCC and SCC associated with number of sunburns, sunscreen use, and use of tanning beds (*n* = 5,783)

	**Melanoma**		**BCC**		**SCC**		
	Crude OR (95% CI)	Adjusted OR^a^ (95% CI)	Crude OR (95% CI)	Adjusted OR^a^ (95% CI)	Crude OR (95% CI)	Adjusted OR^a^ (95% CI)	*P* homogeneity
**Number of sunburns**							
** Before 15 years**							0.77
Never	1.00	1.00	1.00	1.00	1.00	1.00	
1	1.13 (0.67–1.90)	1.10 (0.63–1.91)	1.41 (1.07–1.86)	1.26 (0.96–1.67)	1.40 (0.66–2.99)	1.16 (0.53–2.54)	
2–3	1.64 (1.11–2.43)	1.26 (0.82–1.95)	1.65 (1.32–2.06)	1.48 (1.18–1.86)	3.23 (1.66–6.27)	2.64 (1.24–5.65)	
4–5	2.61 (1.48–4.59)	2.10 (1.15–3.84)	2.42 (1.77–3.30)	2.08 (1.51–2.87)	2.39 (0.95–6.01)	2.27 (0.82–6.28)	
≥6	3.87 (2.26–6.63)	2.69 (1.49–4.88)	2.01 (1.40–2.88)	1.70 (1.16–2.48)	3.47 (1.54–7.84)	2.22 (0.87–5.65)	
*P* for trend	<0.0001	0.007	<0.0001	<0.0001	0.0003	0.02	
** Between 15–25 years**							0.97
Never	1.00	1.00	1.00	1.00	1.00	1.00	
1	1.48 (1.01–2.19)	1.27 (0.83–1.93)	1.26 (1.01–1.58)	1.16 (0.92–1.46)	2.63 (1.47–4.71)	1.98 (1.07–3.68)	
2–3	1.51 (1.04–2.20)	1.25 (0.82–1.89)	1.54 (1.25–1.89)	1.38 (1.12–1.72)	1.89 (1.08–3.30)	1.29 (0.69–2.40)	
4–5	2.08 (1.29–3.34)	1.46 (0.86–2.48)	1.99 (1.52–2.60)	1.70 (1.28–2.24)	3.89 (1.75–8.66)	2.70 (1.15–6.37)	
≥6	3.02 (1.89–4.81)	2.49 (1.49–4.17)	2.35 (1.72–3.22)	1.99 (1.44–2.75)	3.41 (1.50–7.80)	1.91 (0.77–4.75)	
*P* for trend	<0.0001	0.002	<0.0001	<0.0001	0.0003	0.08	
** Since 25 years**							0.60
Never	1.00	1.00	1.00	1.00	1.00	1.00	
1	0.71 (0.48–1.04)	0.69 (0.45–1.05)	1.18 (0.95–1.47)	1.15 (0.92–1.43)	1.27 (0.71–2.30)	1.24 (0.65–2.37)	
2–3	1.10 (0.78–1.54)	1.07 (0.74–1.55)	1.15 (0.94–1.41)	1.08 (0.88–1.32)	1.73 (1.05–2.83)	1.34 (0.76–2.36)	
4–5	1.03 (0.61–1.74)	0.92 (0.51–1.63)	1.48 (1.11–1.99)	1.33 (0.99–1.80)	2.57 (1.17–5.63)	2.53 (1.11–5.76)	
≥6	1.47 (0.93–2.31)	1.39 (0.85–2.29)	1.87 (1.38–2.53)	1.63 (1.19–2.24)	2.31 (1.00–5.35)	1.41 (0.56–3.54)	
*P* for trend	0.13	0.26	0.0001	0.006	0.002	0.07	
**Sunscreen use and level of SPF**							
** Before 15 years**							0.14
No protection	1.00	1.00	1.00	1.00	1.00	1.00	
SPF 8	1.52 (0.83–2.78)	1.28 (0.65–2.51)	0.84 (0.55–1.28)	0.79 (0.52–1.20)	0.79 (0.31–2.01)	0.65 (0.23–1.83)	
SPF 8–15	1.33 (0.61–2.91)	1.27 (0.56–2.86)	0.70 (0.41–1.19)	0.67 (0.39–1.15)	1.77 (0.53–5.92)	1.66 (0.41–6.70)	
SPF >15	0.36 (0.07–1.79)	0.31 (0.06–1.55)	0.58 (0.32–1.02)	0.60 (0.34–1.08)	1.33 (0.48–3.64)	1.48 (0.50–4.36)	
*P* for trend	0.87	0.59	0.02	0.02	0.30	0.33	
** Between 15–25 years**							0.91
No protection	1.00	1.00	1.00	1.00	1.00	1.00	
SPF 8	1.40 (0.92–2.13)	1.25 (0.79–1.98)	1.02 (0.81–1.29)	0.94 (0.74–1.20)	0.78 (0.42–1.44)	0.74 (0.38–1.48)	
SPF 8–15	1.56 (1.08–2.25)	1.20 (0.80–1.80)	1.09 (0.86–1.38)	1.02 (0.80–1.30)	1.28 (0.67–2.45)	0.92 (0.43–1.98)	
SPF 15–30	0.90 (0.54–1.48)	0.72 (0.43–1.19)	1.13 (0.88–1.44)	1.02 (0.80–1.32)	0.59 (0.30–1.14)	0.41 (0.19–0.91)	
SPF >30	1.28 (0.66–2.46)	0.98 (0.47–2.01)	0.54 (0.32–0.91)	0.49 (0.29–0.83)	1.63 (0.64–4.19)	1.16 (0.41–3.26)	
*P* for trend	0.38	0.58	0.57	0.18	0.99	0.38	
** Since 25 years**							0.73
No protection	1.00	1.00	1.00	1.00	1.00	1.00	
SPF 8	1.52 (0.84–2.75)	1.43 (0.75–2.72)	1.08 (0.76–1.54)	1.09 (0.77–1.56)	0.99 (0.40–2.44)	1.12 (0.41–3.04)	
SPF 8–15	1.60 (0.96–2.68)	1.44 (0.82–2.53)	0.95 (0.70–1.29)	0.94 (0.69–1.29)	1.63 (0.70–3.80)	1.69 (0.65–4.36)	
SPF 15–30	1.55 (0.98–2.43)	1.31 (0.79–2.18)	1.19 (0.92–1.55)	1.11 (0.85–1.45)	2.16 (1.13–4.11)	2.42 (1.20–4.88)	
SPF >30	2.09 (1.33–3.26)	1.80 (1.09–2.96)	2.08 (1.63–2.66)	1.91 (1.48–2.46)	1.68 (0.94–3.00)	1.47 (0.76–2.82)	
*P* for trend	0.0007	0.01	<0.0001	<0.0001	0.03	0.15	
**Reapplication of sunscreen**							0.60
Never	1.00	1.00	1.00	1.00	1.00	1.00	
Sometimes	1.68 (1.16–2.42)	1.42 (0.95–2.11)	1.36 (1.11–1.67)	1.31 (1.06–1.62)	1.32 (0.80–2.20)	1.30 (0.72–2.34)	
Always	1.83 (1.18–2.84)	1.51 (0.94–2.43)	1.49 (1.16–1.92)	1.39 (1.07–1.80)	2.07 (1.11–3.86)	2.11 (1.03–4.34)	
*P* for trend	0.008	0.09	0.001	0.008	0.03	0.049	
**Tanning bed use**							0.35
Never	1.00	1.00	1.00	1.00	1.00	1.00	
Ever	1.01 (0.67–1.50)	0.92 (0.60–1.42)	1.10 (0.86–1.42)	1.06 (0.82–1.37)	1.45 (0.75–2.80)	1.72 (0.83–3.59)	

BCC, basal-cell carcinoma; CI, confidence interval; OR, odds ratio; SCC, squamous-cell carcinoma; SPF, sun protection factor.

^a^Adjusted for skin sensitivity to sun exposure, number of naevi, number of freckles, eye color, skin color, and hair color; matched for age, county of birth and education level.

Study population according to the cancer studied: *n* = 1,219 for melanoma; *n* = 3,453 for BCC; *n* = 528 for SCC.

Use of sunscreen with an SPF > 15 before age 15 years was inversely associated with BCC risk (OR 0.60; 95% CI, 0.34–1.08; *P_trend_* = 0.02), but not with melanoma or SCC risks, although no heterogeneity was detected (*P_homogeneity_* = 0.14). Use of sunscreen at ages 15–25 was inversely associated with BCC (SPF > 30: OR 0.49) and SCC (SPF 15–30: OR 0.41) risks, albeit with no evidence of linear relationships.

In contrast, use of high-SPF sunscreen (>30/total sunblock) after age 25 was positively associated with all skin cancer types, more strongly so for BCC (SPF > 30: OR 1.91, *P_trend_* < 0.0001) and melanoma (SPF > 30: OR 1.80, *P_trend_* = 0.01) than for SCC (*P_trend_* = 0.15), although with no evidence of heterogeneity (*P_homogeneity_* = 0.73). In a sensitivity analysis additionally adjusting for various recreational UV exposures (lifetime hours of recreational sun exposure, recreational UV score, number of sunburns since age 25), associations were attenuated but remained statistically significant (eTable 3).

Compared with women who reported that they never reapplied sunscreen during sun exposure, those who reported to always reapply sunscreen had higher risks of all skin cancer types, although statistical significance remained only for BCC and SCC after adjustment (BCC: OR 1.39, *P_trend_* = 0.008; SCC: OR 2.11, *P_trend_* = 0.049). After additional adjustment for other recreational UV exposures, these associations were similar (eTable 3).

We found no statistically significant association between ever use of an indoor tanning device and skin cancer risk, although there was a slightly increased risk of SCC (OR 1.72; 95% CI, 0.83–3.59).

### Lifetime hours of sun exposure and UV score

Total number of hours of sun exposure was positively associated with BCC and SCC risks (Table 2). While hours of residential sun exposure were not significantly associated with skin cancer risk, hours of recreational sun exposure were positively associated with BCC risk (*P_trend_* = 0.001).

**Table 2.** Crude and adjusted ORs for risks of melanoma, BCC and SCC associated with lifetime hours of sun exposure and UV score (*n* = 5,783)

	**Melanoma**		**BCC**		**SCC**		
	Crude OR (95% CI)	Adjusted OR^a^ (95% CI)	Crude OR (95% CI)	Adjusted OR^a^ (95% CI)	Crude OR (95% CI)	Adjusted OR^a^ (95% CI)	*P* homogeneity
**Lifetime hours of sun exposure**							
** Total**							0.51
Tertile 1	1.00	1.00	1.00	1.00	1.00	1.00	
Tertile 2	1.04 (0.77–1.41)	1.07 (0.77–1.50)	1.19 (0.99–1.43)	1.23 (1.02–1.48)	1.70 (1.06–2.71)	1.99 (1.19–3.33)	
Tertile 3	0.90 (0.66–1.24)	0.98 (0.69–1.39)	1.08 (0.90–1.30)	1.20 (0.99–1.46)	1.10 (0.69–1.77)	1.36 (0.80–2.29)	
*P* for trend	0.52	0.91	0.41	0.06	0.69	0.25	
** Residential**							0.55
Tertile 1	1.00	1.00	1.00	1.00	1.00	1.00	
Tertile 2	0.82 (0.61–1.11)	0.89 (0.64–1.24)	0.98 (0.81–1.17)	1.01 (0.84–1.22)	1.31 (0.81–2.10)	1.42 (0.84–2.40)	
Tertile 3	0.79 (0.58–1.09)	0.92 (0.65–1.31)	0.88 (0.73–1.06)	0.94 (0.78–1.14)	1.11 (0.68–1.80)	1.29 (0.76–2.18)	
*P* for trend	0.15	0.62	0.18	0.54	0.71	0.37	
** Recreational**							0.09
Tertile 1	1.00	1.00	1.00	1.00	1.00	1.00	
Tertile 2	0.94 (0.69–1.27)	0.90 (0.64–1.27)	1.26 (1.05–1.51)	1.27 (1.05–1.53)	0.76 (0.47–1.21)	0.87 (0.52–1.47)	
Tertile 3	0.83 (0.61–1.14)	0.88 (0.62–1.24)	1.28 (1.06–1.54)	1.37 (1.13–1.66)	0.88 (0.56–1.38)	1.11 (0.66–1.86)	
*P* for trend	0.25	0.47	0.01	0.001	0.59	0.69	
**UV Score**							
** Total**							0.73
Tertile 1	1.00	1.00	1.00	1.00	1.00	1.00	
Tertile 2	1.24 (0.91–1.69)	1.27 (0.91–1.78)	1.20 (1.00–1.44)	1.26 (1.04–1.52)	1.87 (1.15–3.04)	2.45 (1.39–4.32)	
Tertile 3	1.10 (0.79–1.52)	1.21 (0.85–1.73)	1.15 (0.96–1.39)	1.27 (1.05–1.55)	1.34 (0.84–2.15)	1.69 (1.00–2.87)	
*P* for trend	0.57	0.27	0.14	0.01	0.30	0.09	
** Residential**							0.62
Tertile 1	1.00	1.00	1.00	1.00	1.00	1.00	
Tertile 2	0.90 (0.66–1.22)	0.97 (0.69–1.35)	0.96 (0.80–1.15)	0.98 (0.81–1.18)	1.53 (0.95–2.45)	1.90 (1.12–3.23)	
Tertile 3	0.95 (0.69–1.31)	1.11 (0.78–1.57)	1.03 (0.85–1.23)	1.11 (0.92–1.34)	1.23 (0.76–2.00)	1.47 (0.86–2.52)	
*P* for trend	0.76	0.56	0.78	0.28	0.43	0.16	
** Recreational**							0.67
Tertile 1	1.00	1.00	1.00	1.00	1.00	1.00	
Tertile 2	1.18 (0.87–1.61)	1.20 (0.86–1.69)	1.31 (1.08–1.59)	1.32 (1.08–1.61)	0.88 (0.55–1.41)	0.97 (0.57–1.64)	
Tertile 3	1.17 (0.85–1.61)	1.27 (0.90–1.79)	1.40 (1.16–1.69)	1.47 (1.21–1.79)	1.22 (0.78–1.91)	1.62 (0.96–2.72)	
*P* for trend	0.34	0.18	0.0005	0.0001	0.38	0.07	

BCC, basal-cell carcinoma; CI, confidence interval; OR, odds ratio; SCC, squamous-cell carcinoma; UV, ultraviolet.

^a^Adjusted for skin sensitivity to sun exposure, number of naevi, number of freckles, eye color, skin color and hair color; matched for age, county of birth and education level.

Study population according to the cancer studied: *n* = 1,219 for melanoma; *n* = 3,453 for BCC; *n* = 528 for SCC.

Total UV score was associated with BCC (ORs of 1.26 and 1.27 across tertiles vs. the lowest, *P_trend_* = 0.01) and SCC (ORs of 2.45 and 1.69, *P_trend_* = 0.09) risks. While residential UV score was associated with SCC risk only (OR 1.90 in the second vs. first tertile), recreational UV score was strongly associated with BCC risk (*P_trend_* = 0.0001) and to a lesser extent with SCC risk (*P_trend_* = 0.07). However, no significant heterogeneity was observed between skin cancer subtypes for these exposures.

The associations with UV scores did not substantially differ when considering different types of UV (UVA, UVB, or erythemal UV; data not shown). Stratified analyses according to age at exposure (<25 or ≥25 years) showed stronger associations between total sun exposure before age 25 (hours of sun exposure and UV score) and BCC risk, although there was no statistically significant heterogeneity across strata (eTable 4).

## DISCUSSION

In this nested case-control study within a large prospective cohort of women, we observed that melanoma, BCC, and SCC risks were associated with different patterns of UV exposure, although no statistically significant heterogeneity was observed across skin cancer types. While number of sunburns before age 25 was strongly associated with melanoma risk, recreational sun exposure was more strongly related to BCC, and total and residential sun exposure was more strongly related to SCC. In addition, while the use of high-SPF sunscreen before age 25 was associated with lower BCC risk, use since age 25 and reapplication of sunscreen were associated with higher risks of all three types of skin cancer.

### Sunburn, sunscreen use, and indoor tanning

In our study, the number of sunburns was positively associated with skin cancer risk. Although ORs were attenuated after adjustment for phenotypic factors, associations remained statistically significant, indicating that a history of sunburns is a risk factor for melanoma, BCC, and SCC independently of the number of naevi and freckles and skin, hair, and eye color. However, we observed no association between number of sunburns occurring after age 25 years and melanoma risk. This is consistent with several studies suggesting that childhood and adolescence are the most sensitive time periods for exposure to sunburns for this cancer,^14^^,^^15^^,^^23^^–^^25^ although other studies have reported a positive association between a history of sunburn at any age and melanoma.^26^^–^^28^

Although lifetime number of severe sunburns has been associated with keratinocyte cancer risk,^16^ few studies explored timing of exposure. A case-control study in the Netherlands reported that the recall of painful sunburns before age 20 years was associated with increased risks of SCC, nodular BCC, and multifocal superficial BCC.^29^ In a Canadian case-control study, a history of severe sunburn in childhood (but not lifetime sunburns) was associated with higher BCC risk.^30^ In our study, number of sunburns at any age was associated with BCC, and a linear relationship was apparent only for sunburns occurring before age 15 for SCC risk.

Data from randomized controlled trials suggest protective effects of sunscreen use on melanoma and SCC risks.^31^^,^^32^ More generally, a recent review of randomized controlled trials suggested that the evidence, though limited, supports beneficial effects of sunscreen application on the occurrence of skin cancers.^33^ However, findings from observational studies have been inconsistent,^33^ with some studies showing an inverse association^25^^,^^34^^–^^37^ and others suggesting a positive association.^38^^,^^39^ This heterogeneity may be explained by the fact that non-randomized studies are unable to distinguish the main determinants of sunscreen use from those of skin cancer because they are similar^40^ (eg, sun-sensitive phenotypes). For example, it has been proposed that sunscreen use may encourage prolonged sun exposure duration because it delays sunburn occurrence,^41^ especially when exposure is associated with intention to tan or stay in the sun.^42^ In our study, additional adjustment for recreational UV exposure variables had little impact on the results. However, other factors could interact in these relationships, and residual confounding cannot be ruled out.

Recently, two meta-analyses showed that tanning bed use was associated with significantly increased risks of melanoma, BCC, and SCC.^43^^,^^44^ We found a positive association with SCC risk only, although the OR did not reach statistical significance. Because tanning beds started to be commonly used in the 1980s, it is likely that the women in our study population, who were aged 57–85 years when responding to the questionnaire in 2008, were not exposed at young ages. Young age at first exposure, which was not available in our data, has indeed been associated with high risks of both melanoma^43^^,^^45^^,^^46^ and keratinocyte cancers.^44^^,^^47^

### Lifetime hours of sun exposure and UV score

In our study, both total number of hours of sun exposure and total UV score increased the risk of developing BCC and SCC, which is consistent with previous studies.^13^^,^^29^ Although we found no association with melanoma, a positive association between total sun exposure and melanoma risk (RR 1.34; 95% CI, 1.02–1.77) was reported in a meta-analysis.^15^ While hours of residential sun exposure were not associated with skin cancer risk in our study, the residential UV score was associated with an increased SCC risk, consistent with the hypothesis of SCC being associated with long-term, cumulative exposure.^9^^,^^16^^,^^18^^,^^19^ However, English et al showed that SCC was more strongly related to total sun exposure than to residential sun exposure,^3^ which was also the case in our study.

Substantial evidence suggests that recreational sun exposure is the major causal factor of BCC.^9^^,^^12^ Consistently, in our study, hours of recreational sun exposure and the recreational UV score were associated with increased BCC risk, and BCC risk was more strongly associated with recreational exposure than with total (both residential and recreational) exposure. Recreational sun exposure is also known to be strongly associated with melanoma,^15^^,^^16^^,^^25^^,^^48^ which was not evidenced through lifetime hours of recreational sun exposure or recreational UV score in our analyses.

While the damaging effects of UV exposure on the skin are thought to be caused by direct cellular damage and alterations in immunologic function, there is uncertainty regarding the mechanism by which each skin cancer type develops in relation to different types of solar exposure (frequent or intermittent).^17^ Several studies suggested that childhood and adolescence may be sensitive periods regarding BCC risk in adulthood.^30^^,^^49^^,^^50^ However, we found no statistically significant heterogeneity in associations between total sun exposure and BCC risk across strata of age of exposure, although associations were stronger before age 25.

While UVA and UVB are both important in skin cancer development,^17^ comparisons of skin cancer data from Norway, Australia, and New Zealand suggest that SCC and BCC are mainly related to annual solar UVB, while UVA plays a larger role in melanoma.^51^ However, our analyses by UV type showed similar trends for UVA, UVB, and erythemal UV across skin cancer types.

Several limitations should be considered in the interpretation of our findings. First, because UV exposures were collected retrospectively and after diagnosis for cases, our results are subject to recall bias — a bias inherent to the case-control design that may lead to differential misclassification and biased risk estimates, especially considering the time interval between past exposures and study inception.^52^ In particular, this bias may have led to a spurious positive association between sunscreen use since age 25 and skin cancer risk, and potentially to a null association between tanning bed use and skin cancer. Second, because sunscreen is likely used by at-risk subjects, an indication bias may have occurred and could again potentially have led to a spurious positive association between sunscreen use after age 25 and skin cancer — although inverse associations with use before age 25 were observed. In addition, when asked about sunscreen use since age 25, it is possible that cases have reported their most frequent use of sunscreen after diagnosis, which could also explain part of the positive association between sunscreen use and skin cancer risk. Third, selection bias could be a concern, given the lower response rate in controls than in cases; however, apart from case-control status, other characteristics were similar between respondents and non-respondents. Finally, because skin cancer cases who died prior to the study could not be included as participants, a survival bias may have occurred, which may lead to an underestimation of associations. However, given the low numbers of cases deceased before the study, a substantial impact of this bias on the findings is unlikely. Despite these limitations, our study has several strengths, particularly its high study response rate, the fact that cases and controls were sampled from the same source population, the ability to control for pigmentary traits, and the availability of summary variables quantifying sun exposure over lifetime, using different types of UV radiation, and including data on sun protection. We were also able to compare UV exposure patterns across skin cancer types, an analysis that has rarely been possible in previous research.

In conclusion, our findings suggest that skin cancer types are associated with different patterns of sun exposure. While number of sunburns before age 25 was strongly associated with risk of melanoma, recreational sun exposure was more strongly related to BCC, and total and residential sun exposure was more strongly related to SCC. Avoiding sunburns at any age, and especially at younger ages, is strongly recommended to prevent skin cancer, and protective measures should be taken against all types of UV exposure (recreational or residential) to minimize the risk of developing any form of skin cancer.

## APPENDIX A. SUPPLEMENTARY DATA

eMethods 1. Detailed calculation of lifetime number of hours of sun exposure and UV score

eTable 1. Characteristics of respondents and non-respondents, E3N-SunExp study

eTable 2. Characteristics of the study population according to skin cancer status in the E3N-SunExp study

eTable 3. ORs for risks of melanoma, BCC, and SCC associated with sunscreen use after additional adjustment for UV exposures

eTable 4. ORs for risks of melanoma, BCC, and SCC associated with UV exposures according to age at exposure
