# Supplementary material for: Patterns of Ultraviolet Radiation Exposure and Skin Cancer Risk: the E3N-SunExp Study
Source: J Epidemiol. 2018 Jan 5;28(1):27–33. doi: 10.2188/jea.JE20160166 (PMC5742376; doi:10.2188/jea.JE20160166)
Supplement: Supplementary file 1 [file je-28-027-s001.pdf]

eMethods 1. Detailed calculation of lifetime number of hours of sun exposure and UV score

Lifetime number of hours of sun exposure

To calculate the total number of hours of recreational sun exposure, we multiplied the number of weeks of holidays and weekends/days off in each location by the amount of time spent in the sun in those locations. Weights were assigned to each category of time spent in the sun as follows: 0.5, 2.5, and 6 for “1 hour/day or less”, “2-3 hours/day” and “4 hours/day or more”, respectively. The number of hours of residential sun exposure was calculated in a similar manner, additionally subtracting number of weeks of holidays and weekends/days off from the calculation (considering a total of 2.5 days per week for weekends/days off). In order to take into account sun protection level in the calculations, we assigned the following weights to each category of sun protection: 0.33, 0.66, and 1 for “never or rarely”, “sometimes” and “often or always”, respectively, and we applied these weights to the calculations. To obtain a total lifetime number of hours of sun exposure, we summed total numbers of recreational and residential hours of sun exposure.

UV score

To calculate a score based on the estimated doses of different types of UV radiation, we first extracted data on latitude and longitude of each residence and holiday locations reported by the study participants using publicly available databases (latitude-longitude geocoding database of the French National Institute of Statistics and Economic Studies (INSEE); Geographic Directory of French Counties (RGC) database of the French National Geography Institute (IGN)).

We then linked this geocoding information to an international database containing UV irradiance data according to latitude and longitude,[1] which is available via the Internet-based Solar Radiation Data (SoDa) service (MINES ParisTech website: www.soda-is.com).[2] Briefly, the UV data contained in this database resulted from a clear-sky model with modifications for cloud effects. We used the default inputs to the model proposed by the SoDa service[1]: total atmospheric column content of ozone and water vapour, Linke turbidity factor, Angström coefficient for aerosols and cloud cover, ground albedo, and surface elevation above mean sea level. These variables came from different sources covering various periods: 1996–2000 for ozone, Linke turbidity factor, and cloud cover; 1987–1996 for water vapour; and 1981–1990 for Angström coefficient.[1,3] We thus obtained monthly means of daily sums (daily doses) for total UV, UVA, UVB, and erythemal UV in J/m^2. The modelled UV values were within 10% of measurements taken at Reading in the United Kingdom.[1]

By linkage to the SoDa data, we obtained mean daily UV doses for spring/summer (April–September) and autumn/winter (October–March) for each residence and holiday location, which we used to calculate UV exposure scores for each participant.

We first calculated a recreational UV score by summing UV doses in holiday locations over lifetime (weighted by holiday duration in each location) and UV dose over weekends/days off. A residential UV score per year of age was then calculated by summing autumn/winter and spring/summer score calculations of work/school days only, and by taking into account holiday duration for each year of age. The total UV score for each year of age was the sum of the recreational and residential scores. Lifetime hours of sun exposure and UV scores were categorized in tertiles for the analyses.

There were high correlations between total number of hours of sun exposure and total UV score ($r_{Spearman}$=0.86), residential number of hours of sun exposure and residential UV score (r=0.84) and recreational number of hours of sun exposure and recreational UV score (r=0.76).

1. Kift, R., Webb, A. R., Page, J., Rimmer, J. & Janjai, S. A Web-based Tool for UV lrradiance Data: Predictions for European and Southeast Asian Sites. *Photochem. Photobiol.* **82,** 579–586 (2006).
2. Gschwind, B., Ménard, L., Albuisson, M. & Wald, L. Converting a successful research project into a sustainable service: The case of the SoDa Web service. *Environ. Model. Softw.* **21,** 1555–1561 (2006).
3. Remund, J. Advanced parameters WP 5.2b: chain of algorithms: short- and longwave radiation with associated temperature prediction resources. IN Report to the European Commission. (2002).

eTable 1. Characteristics of respondents and non-respondents, E3N-SunExp study

	Respondents		Non-respondents		
	N	% / Mean (SD)	N	% / Mean (SD)	p-value
**Age at the time of the questionnaire (years)**	5783	68.9 (6.4)	1470	69.8 (7.0)	<0.001
**Skin cancer status**					<0.001
Cases	1566	27.1	246	16.7	
Controls	4217	72.9	1224	83.3	
**Education level (years)**					<0.001
<12	484	8.4	181	12.3	
12-15	2958	51.2	716	48.7	
≥15	2109	36.5	514	35.0	
Missing	232	4.0	59	4.0	
**Skin sensitivity to sun exposure**					0.89
High	1701	29.5	424	28.8	
Moderate	2689	46.5	680	46.3	
Low	1279	22.1	337	22.9	
Missing	108	1.9	29	2.0	
**Number of naevi**					0.45
Very many	695	12.0	157	10.7	
Many	2387	41.3	609	41.4	
A few/None	2593	44.8	680	46.3	
Missing	108	1.9	24	1.6	
**Number of freckles**					0.63
Very many	352	6.1	77	5.2	
Many	1752	30.3	430	29.3	
A few	1420	24.6	367	25.0	
None	2096	36.2	553	37.6	
Missing	163	2.8	43	2.9	
**Hair colour**					0.59
Red	126	2.2	25	1.7	
Blond	602	10.4	164	11.2	
Chestnut	3426	59.2	885	60.2	
Brown/Dark	1527	26.4	373	25.4	
Missing	102	1.8	23	1.6	
**Skin colour**					0.92
Very fair/fair	3411	59.0	872	59.3	
Medium/Olive/Dark	2261	39.1	572	38.9	
Missing	111	1.9	26	1.8	
**Family history of skin cancer**					0.15
No	5700	98.6	1456	99.0	
Yes	83	1.4	14	1.0	

SD. standard deviation.

eTable 2. Characteristics of the study population according to skin cancer status in the E3N-SunExp study

	Melanoma		BCC		SCC		Controls	
	N	% / Mean (SD)	N	% / Mean (SD)	N	% / Mean (SD)	N	%
**Age at diagnosis (years)**	366	57 (7)	1027	59 (7)	165	63 (8)		
**Education level (years)**								
<12	35	9.6	92	9.0	11	6.7	293	8.1
12-15	194	53.0	511	49.8	84	50.9	1877	51.5
≥15	124	33.9	381	37.1	64	38.8	1328	36.5
Missing	13	3.6	43	4.2	6	3.6	144	4.0
**Skin sensitivity to sun exposure**								
High	144	39.3	337	32.8	69	41.8	1022	28.1
Moderate	169	46.2	493	48.0	75	45.5	1677	46.1
Low	49	13.4	173	16.9	19	11.5	881	24.2
Missing	4	1.1	24	2.3	2	1.2	62	1.7
**Number of naevi**								
Very many	86	23.5	163	15.9	17	10.3	372	10.2
Many	174	47.5	488	47.5	58	35.2	1454	39.9
A few/None	100	27.3	354	34.5	88	53.3	1756	48.2
Missing	6	1.6	22	2.1	2	1.2	60	1.7
**Number of freckles**								
Very many	39	10.7	90	8.8	28	17.0	164	4.5
Many	145	39.6	354	34.5	54	32.7	1040	28.6
A few	78	21.3	228	22.2	36	21.8	929	25.5
None	97	26.5	328	31.9	44	26.7	1405	38.6
Missing	7	1.9	27	2.6	3	1.8	104	2.9
**Hair colour**								
Red	21	5.7	31	3.0	5	3.0	63	1.7
Blond	60	16.4	114	11.1	11	6.7	353	9.7
Chestnut	221	60.4	609	59.3	112	67.9	2139	58.7
Brown/Dark	61	16.7	255	24.8	33	20.0	1026	28.2
Missing	3	0.8	18	1.8	4	2.4	61	1.7
**Skin colour**								
Very fair/fair	261	71.3	644	62.7	124	75.2	2074	57.0
Medium/Olive/Dark	100	27.3	361	35.2	37	22.4	1507	41.4
Missing	5	1.4	22	2.1	4	2.4	61	1.7
**Eye colour**								
Blue/Grey	118	32.2	298	29.0	49	29.7	1010	27.7
Green/Hazel	186	50.8	513	50.0	91	55.2	1734	47.6
Brown/Black	58	15.9	206	20.1	25	15.2	868	23.8
Missing	4	1.1	10	1.0	0	0.0	30	0.8

eTable 2. Characteristics of the study population according to skin cancer status in the E3N-SunExp study

	Melanoma		BCC		SCC		Controls	
	N	% / Mean (SD)	N	% / Mean (SD)	N	% / Mean (SD)	N	%
**Number of sunburns**								
**Before 15 years**								
Never	119	32.5	362	35.3	53	32.1	1720	47.2
1	24	6.6	96	9.4	12	7.3	325	8.9
2-3	54	14.8	151	14.7	29	17.6	444	12.2
4-5	32	8.7	79	7.7	8	4.9	148	4.1
≥6	39	10.7	70	6.8	16	9.7	162	4.5
Don't know	64	17.5	160	15.6	23	13.9	407	11.2
Missing	34	9.3	109	10.6	24	14.6	436	12.0
**Between 15-25 years**								
Never	75	20.5	236	23.0	33	20.0	1151	31.6
1	73	20.0	176	17.1	34	20.6	686	18.8
2-3	94	25.8	279	27.2	39	23.6	893	24.5
4-5	43	11.8	128	12.5	18	10.9	298	8.2
≥6	54	14.8	100	9.7	15	9.1	221	6.1
Don't know	10	2.7	44	4.3	13	7.9	116	3.2
Missing	17	4.6	64	6.2	13	7.9	277	7.6
**Since 25 years**								
Never	142	38.8	379	36.9	53	32.1	1511	41.5
1	47	12.8	161	15.7	20	12.1	565	15.5
2-3	77	21.0	210	20.5	41	24.9	731	20.1
4-5	34	9.3	93	9.1	17	10.3	248	6.8
≥6	40	10.9	89	8.7	11	6.7	207	5.7
Don't know	6	1.6	21	2.0	10	6.1	65	1.8
Missing	20	5.5	74	7.2	13	7.9	315	8.7
**Sunscreen use and level of SPF**								
**Before 15 years**								
No protection	217	59.3	678	66.0	104	63.0	2190	60.1
SPF 8	19	5.2	34	3.3	6	3.6	130	3.6
SPF 8-15	10	2.7	18	1.8	5	3.0	89	2.4
SPF >15	2	0.6	14	1.4	6	3.6	84	2.3
Don't know	93	25.4	215	20.9	33	20.0	791	21.7
Missing	25	6.8	68	6.6	11	6.7	358	9.8
**Between 15-25 years**								
No protection	123	33.6	389	37.9	72	43.6	1317	36.2
SPF 8	57	15.6	142	13.8	16	9.7	488	13.4
SPF 8-15	64	17.5	140	13.6	21	12.7	436	12.0
SPF 15-30	32	8.7	129	12.6	13	7.9	392	10.8
SPF >30	14	3.8	20	2.0	9	5.5	130	3.6

eTable 2. Characteristics of the study population according to skin cancer status in the E3N-SunExp study

	Melanoma		BCC		SCC		Controls	
	N	% / Mean (SD)	N	% / Mean (SD)	N	% / Mean (SD)	N	%
Don't know	53	14.5	153	14.9	25	15.2	580	15.9
Missing	23	6.3	54	5.3	9	5.5	299	8.2
**Since 25 years**								
No protection	35	9.6	120	11.7	19	11.5	560	15.4
SPF 8	26	7.1	68	6.6	10	6.1	299	8.2
SPF 8-15	50	13.7	101	9.8	14	8.5	481	13.2
SPF 15-30	92	25.1	233	22.7	44	26.7	907	24.9
SPF >30	133	36.3	424	41.3	63	38.2	1012	27.8
Don't know	16	4.4	50	4.9	8	4.9	225	6.2
Missing	14	3.8	31	3.0	7	4.2	158	4.3
**Reapplication of sunscreen**								
Never	41	11.2	132	12.9	25	15.2	588	16.1
Sometimes	224	61.2	608	59.2	83	50.3	2033	55.8
Always	73	20.0	192	18.7	30	18.2	585	16.1
Don't know	5	1.4	9	0.9	3	1.8	50	1.4
Missing	23	6.3	86	8.4	24	14.6	386	10.6
**Tanning bed use**								
No	326	89.1	925	90.1	149	90.3	3291	90.4
Yes	40	10.9	102	9.9	16	9.7	351	9.6
**Lifetime hours of sun exposure**								
**Total**[a]								
Tertile 1	132	36.1	311	30.3	41	24.9	1228	33.7
Tertile 2	126	34.4	363	35.4	69	41.8	1170	32.1
Tertile 3	108	29.5	353	34.4	55	33.3	1244	34.2
**Residential**[a]								
Tertile 1	141	38.5	347	33.8	45	27.3	1191	32.7
Tertile 2	117	32.0	344	33.5	59	35.8	1197	32.9
Tertile 3	108	29.5	336	32.7	61	37.0	1254	34.4
**Recreational**[a]								
Tertile 1	138	37.7	292	28.4	56	33.9	1217	33.4
Tertile 2	119	32.5	355	34.6	51	30.9	1203	33.0
Tertile 3	109	29.8	380	37.0	58	35.2	1222	33.6
**UV Score**								
**Total**[b]								
Tertile 1	126	34.4	303	29.5	41	24.9	1236	33.9
Tertile 2	127	34.7	362	35.3	67	40.6	1165	32.0
Tertile 3	113	30.9	362	35.3	57	34.6	1241	34.1
**Residential**[b]								
Tertile 1	132	36.1	343	33.4	42	25.5	1208	33.2

eTable 2. Characteristics of the study population according to skin cancer status in the E3N-SunExp study

		Melanoma		BCC		SCC		Controls	
		N	% / Mean (SD)	N	% / Mean (SD)	N	% / Mean (SD)	N	%
	Tertile 2	115	31.4	325	31.7	65	39.4	1198	32.9
	Tertile 3	119	32.5	359	35.0	58	35.2	1236	33.9
**Recreational[b]**									
	Tertile 1	126	34.4	300	29.2	56	33.9	1221	33.5
	Tertile 2	116	31.7	335	32.6	49	29.7	1223	33.6
	Tertile 3	124	33.9	392	38.2	60	36.4	1198	32.9

BCC, basal-cell carcinoma; SCC, squamous-cell carcinoma; SD, standard deviation; SPF, sun protection factor; UV, ultraviolet.

[a]Cut-off points for tertiles were as follows: 16 799.12 and 26 774.13 for total number of hours of sun exposure; 4230 and 9947.16 for hours of residential sun exposure; and 10 156.01 and 16 365.05 for hours of recreational sun exposure.

[b]Cut-off points for tertiles were as follows: 20 003 924.57 and 33 338 466.05 for total UV score; 4 668 326.93 and 11 121 039.83 for residential UV score; and 10 861 934.69 and 17 783 706.71 for recreational UV score.

eTable 3. ORs for risks of melanoma, BCC, and SCC associated with sunscreen use after additional adjustment for UV exposures

	Melanoma	BCC	SCC
	OR[a] (95% CI)	OR[a] (95% CI)	OR[a] (95% CI)
**Level of SPF since 25 years**			
No protection	1.00	1.00	1.00
SPF 8	1.39 (0.72-2.69)	1.04 (0.72-1.49)	0.98 (0.33-2.90)
SPF 8-15	1.46 (0.81-2.64)	0.92 (0.67-1.26)	1.47 (0.52-4.16)
SPF 15-30	1.31 (0.77-2.21)	1.08 (0.83-1.42)	2.40 (1.13-5.10)
SPF >30	1.83 (1.09-3.07)	1.96 (1.51-2.53)	1.45 (0.73-2.87)
P for trend	0.02	<0.0001	0.12
**Reapplication of sunscreen**			
Never	1.00	1.00	1.00
Sometimes	1.38 (0.91-2.08)	1.30 (1.06-1.61)	1.22 (0.65-2.29)
Always	1.50 (0.92-2.46)	1.43 (1.10-1.85)	2.13 (1.00-4.54)
P for trend	0.12	0.004	0.06

BCC, basal-cell carcinoma; CI, confidence interval; OR, odds ratio; SCC, squamous-cell carcinoma; SPF, sun protection factor.

[a]Adjusted for skin sensitivity to sun exposure, number of naevi, number of freckles, eye colour, skin colour, hair colour, number of recreational sun exposure, recreational UV score and sunburns >25 years; matched for age, county of birth and education level.

eTable 4. ORs for risks of melanoma, BCC, and SCC associated with UV exposures according to age at exposure

	Melanoma			BCC			SCC		
	<25 years	≥25 years		<25 years	≥25 years		<25 years	≥25 years	
	Adjusted OR[a] (95% CI)	Adjusted OR[a] (95% CI)	*P* homogeneity	Adjusted OR[a] (95% CI)	Adjusted OR[a] (95% CI)	*P* homogeneity	Adjusted OR[a] (95% CI)	Adjusted OR[a] (95% CI)	*P* homogeneity
**Lifetime hours of sun exposure**									
**Total**									
Tertile 1	1.00	1.00	0.85	1.00	1.00	0.19	1.00	1.00	0.72
Tertile 2	0.98 (0.71-1.35)	1.03 (0.73-1.44)		1.10 (0.91-1.33)	1.11 (0.92-1.34)		1.55 (0.90-2.65)	2.05 (1.16-3.62)	
Tertile 3	0.96 (0.69-1.34)	0.82 (0.58-1.17)		1.32 (1.09-1.59)	1.02 (0.84-1.24)		1.53 (0.90-2.62)	1.21 (0.70-2.08)	
P for trend	0.81	0.30		0.005	0.82		0.13	0.70	
**Residential**									
Tertile 1	1.00	1.00	0.72	1.00	1.00	0.64	1.00	1.00	0.95
Tertile 2	0.75 (0.53-1.05)	0.82 (0.58-1.16)		0.99 (0.82-1.19)	0.87 (0.71-1.05)		0.97 (0.58-1.60)	1.43 (0.84-2.45)	
Tertile 3	0.69 (0.49-0.97)	0.85 (0.60-1.20)		0.98 (0.81-1.18)	0.85 (0.71-1.03)		1.10 (0.64-1.87)	1.26 (0.75-2.13)	
P for trend	0.03	0.35		0.80	0.11		0.74	0.43	
**Recreational**									
Tertile 1	1.00	1.00	0.41	1.00	1.00	0.11	1.00	1.00	0.95
Tertile 2	1.15 (0.83-1.61)	1.04 (0.74-1.46)		1.32 (1.09-1.59)	1.09 (0.90-1.31)		1.03 (0.61-1.71)	1.48 (0.88-2.48)	
Tertile 3	1.16 (0.83-1.64)	0.83 (0.59-1.19)		1.54 (1.27-1.86)	1.16 (0.96-1.40)		1.12 (0.67-1.85)	1.26 (0.75-2.11)	
P for trend	0.39	0.31		<0.0001	0.13		0.67	0.38	
**UV Score**									
**Total**									
Tertile 1	1.00	1.00	0.58	1.00	1.00	0.21	1.00	1.00	0.76
Tertile 2	1.34 (0.97-1.84)	1.04 (0.74-1.46)		1.12 (0.92-1.36)	1.11 (0.92-1.33)		1.55 (0.90-2.68)	2.19 (1.26-3.80)	
Tertile 3	1.17 (0.83-1.63)	0.90 (0.63-1.28)		1.38 (1.14-1.68)	1.08 (0.89-1.31)		1.85 (1.06-3.21)	1.45 (0.86-2.47)	
P for trend	0.34	0.59		0.001	0.41		0.03	0.23	
**Residential**									
Tertile 1	1.00	1.00	0.96	1.00	1.00	0.73	1.00	1.00	0.68
Tertile 2	0.94 (0.67-1.32)	1.21 (0.86-1.70)		1.02 (0.84-1.23)	0.90 (0.74-1.09)		1.76 (1.03-3.00)	1.66 (0.98-2.81)	
Tertile 3	1.09 (0.78-1.54)	1.02 (0.72-1.44)		1.13 (0.93-1.37)	1.01 (0.84-1.22)		1.67 (0.96-2.91)	1.19 (0.70-2.04)	
P for trend	0.62	0.94		0.22	0.89		0.08	0.58	

**Recreational**									
Tertile 1	1.00	1.00	0.53	1.00	1.00	1.00	1.00	1.00	0.98
Tertile 2	1.30 (0.91-1.87)	1.14 (0.81-1.60)		1.07 (0.88-1.31)	1.25 (1.03-1.51)		0.85 (0.50-1.44)	1.19 (0.68-2.09)	
Tertile 3	1.30 (0.91-1.85)	0.97 (0.69-1.37)		1.36 (1.12-1.65)	1.38 (1.14-1.67)		1.47 (0.87-2.48)	1.34 (0.80-2.25)	
P for trend	0.15	0.88		0.002	0.001		0.17	0.27	

BCC, basal-cell carcinoma; CI, confidence interval; OR, odds ratio; SCC, squamous-cell carcinoma; SPF, sun protection factor.

[a]Adjusted for skin sensitivity to sun exposure, number of naevi, number of freckles, eye colour, skin colour and hair colour; matched for age, county of birth and education level.

Study population according to the cancer studied: *n*=1219 for melanoma; *n*=3453 for BCC; *n*=528 for SCC.
